# Safety and immunogenicity of a recombinant protein RBD fusion heterodimer vaccine against SARS-CoV-2

**DOI:** 10.1038/s41541-023-00736-5

**Published:** 2023-09-29

**Authors:** Lorna Leal, Judit Pich, Laura Ferrer, Jocelyn Nava, Ruth Martí-Lluch, Ignasi Esteban, Edwards Pradenas, Dàlia Raïch-Regué, Antoni Prenafeta, Karla Escobar, Carmen Pastor, Marc Ribas-Aulinas, Benjamin Trinitè, Jordana Muñoz-Basagoiti, Gemma Domenech, Bonaventura Clotet, Júlia Corominas, Aida Corpes-Comes, Carme Garriga, Antonio Barreiro, Nuria Izquierdo-Useros, Joan Albert Arnaiz, Alex Soriano, José Ríos, Marga Nadal, Montserrat Plana, Julià Blanco, Teresa Prat, Elia Torroella, Rafel Ramos, HIPRA-HH-1 study group, Eva Bonfill, Omar Anagua, Faisury Caicedo, Clara Castán, Fauno Guazina, Sara Messeguer, Marta Aldea, Anna Vilella, Sandra Serrano, Lorna Leal, Judit Pich, Jocelyn Nava, Karla Escobar, Joan Albert Arnaiz, Alex Soriano, José Ríos, Teresa Botta, Ignasi Esteban, Carmen Pastor, Montserrat Plana, Gemma Domenech, Silvia Marfil, Carla Rovirosa, Raquel Ortiz, Daniel Perez-Zsolt, Marçal Gallemí, Edwards Pradenas, Dàlia Raïch-Regué, Benjamin Trinité, Jordana Muñoz-Basagoiti, Bonaventura Clotet, Nuria Izquierdo-Useros, Julià Blanco, Marina González del Río, Ruth Martí-Lluch, Marc Ribas-Aulinas, Aida Corpes-Comes, Marga Nadal, Rafel Ramos, Luís González, Manuel Cañete, Laia Madrenas, Alexandra Moros, Irina Güell, Laura Ferrer, Antoni Prenafeta, Júlia Corominas, Carme Garriga, Antonio Barreiro, Teresa Prat, Elia Torroella

**Affiliations:** 1 Infectious Diseases Department, Hospital Clínic Barcelona, Barcelona, Spain; 2 Institut d’Investigacions Biomèdiques August Pi i Sunyer (IDIBAPS), Barcelona, Spain; 3 Faculty of Medicine, Universitat de Barcelona, Barcelona, Spain; 4 Clinical Trials Unit (CTU), Hospital Clínic Barcelona, Barcelona, Spain; 5 HIPRA. Avenida La Selva, 135, 17170 Amer (Girona), Spain; 6 Institut Universitari d’‘Investigació en Atenció Primària Jordi Gol (IDIAP Jordi Gol), Girona, Catalonia Spain; 7 Girona Biomedical Research Institute (IDIBGI), Salt, Girona, Spain; 8 IrsiCaixa AIDS Research Institute, Hospital Universitari Germans Trias i Pujol, Campus Can Ruti, Badalona, Spain; 9 Medical Statistics Core Facility, Institut d’Investigacions Biomédiques August Pi i Sunyer (IDIBAPS), Barcelona, Spain; 10 Chair of Infectious Diseases and Immunity, Faculty of Medicine, Universitat de Vic-Universitat Central de Catalunya (uVic-UCC), Vic, Spain; 11 CIBER Enfermedades Infecciosas (CIBERINFEC), Instituto de Salud Carlos III, Madrid, Spain; 12 Department of Clinical Pharmacology, Hospital Clinic Barcelona, Barcelona, Spain; 13 Biostatistics Unit, Faculty of Medicine, Universitat Autònoma de Barcelona, Barcelona, Spain; 14 Germans Trias i Pujol Research Institute (IGTP), Campus Can Ruti, Badalona, Barcelona, Spain; 15 Department of Medical Sciences, School of Medicine, University of Girona, Girona, Spain; 16 Hospital Clinic Barcelona, Insitut d’Investigacions Biomèdiques August Pi i Sunyer (IDIBAPS), Universitat de Barcelona, Barcelona, Spain; 17 IrsiCaixa AIDS Research Institute, Badalona, Spain; 18 Institut Universitari d’Investigació en Atenció Primària Jordi Gol (IDIAP Jordi Gol), Biomedical Research Institute (IdIBGi), Girona, Spain; 19 HIPRA. Avenida la Selva, 135, 17170, Amer (Girona), Spain

**Keywords:** Translational research, Randomized controlled trials, Drug development

## Abstract

In response to COVID-19 pandemic, we have launched a vaccine development program against SARS-CoV-2. Here we report the safety, tolerability, and immunogenicity of a recombinant protein RBD fusion heterodimeric vaccine against SARS-CoV-2 (PHH-1V) evaluated in a phase 1-2a dose-escalation, randomized clinical trial conducted in Catalonia, Spain. 30 young healthy adults were enrolled and received two intramuscular doses, 21 days apart of PHH-1V vaccine formulations [10 µg (*n* = 5), 20 µg (*n* = 10), 40 µg (*n* = 10)] or control [BNT162b2 (*n* = 5)]. Each PHH-1V group had one safety sentinel and the remaining participants were randomly assigned. The primary endpoint was solicited events within 7 days and unsolicited events within 28 days after each vaccination. Secondary endpoints were humoral and cellular immunogenicity against the variants of concern (VOCs) alpha, beta, delta and gamma. All formulations were safe and well tolerated, with tenderness and pain at the site of injection being the most frequently reported solicited events. Throughout the study, all participants reported having at least one mild to moderate unsolicited event. Two unrelated severe adverse events (AE) were reported and fully resolved. No AE of special interest was reported. Fourteen days after the second vaccine dose, all participants had a >4-fold change in total binding antibodies from baseline. PHH-1V induced robust humoral responses with neutralizing activities against all VOCs assessed (geometric mean fold rise at 35 days *p* < 0.0001). The specific T-cell response assessed by ELISpot was moderate. This initial evaluation has contributed significantly to the further development of PHH-1V, which is now included in the European vaccine portfolio.

ClinicalTrials.gov Identifier NCT05007509

EudraCT No. 2021-001411-82

## Introduction

The coronavirus disease 2019 (COVID-19) caused by the Severe Acute Respiratory Syndrome Coronavirus 2 (SARS-CoV-2) has overwhelmed global health and has led us to social and economic exhaustion. Consequently, there has been an unprecedented effort to develop different types of vaccines against this virus that have been remarkable^1^ and relevant in preventing severe disease and minimizing death. These first vaccines have based their antigen on a stabilized trimeric structure of the Spike glycoprotein (S) from the ancestral strain isolated in earliest identified infections. However, viral sequence evolution leading to emergence of variants has impacted on vaccine effectiveness, which has entailed an overexertion in the attempt to adapt vaccines. In May 2023 the World Health Organization (WHO) declared the end of the emergency of the pandemic^2^ and recommended to integrate COVID-19 vaccination into life course vaccination programs. Nevertheless, there is still an unmet need to cover vaccination worldwide, which is low in some regions and highly inequitable^3,4^, while facing an increasingly transmissible virus that remains a health threat and still places a burden on health care systems. We need more vaccines available to overcome all these challenges.

In response to this pandemic and to contribute to the solution we have launched a vaccine development program against SARS-CoV-2. PHH-1V vaccine is a SARS-CoV-2 recombinant spike (S) protein receptor binding domain (RBD) fusion heterodimer containing the B.1.351 (beta) and B.1.1.7 (alpha) variants and co-formulated with an oil-in-water emulsion adjuvant named SQBA, produced by the Sponsor HIPRA. The RBD is a key functional component within the S protein that is responsible for binding SARS-CoV-2 to its cell receptor^5^, and it is one of the main targets for neutralizing antibodies^6^. Using just the RBD region as an antigen has the advantage of focusing immunity to key protective determinants^6,7^, which is supported as the main biomarker of protection^8,9^. Another advantage is that these antigens can be scaled and produced faster and easier compared to the entire S protein or its subunits (S1, S2)^10^. Monomeric RBD has a limited immunogenicity possibly related to its small molecular size and the mixed forms of multiple complexes. To solve this, we have produced a highly purified RBD heterodimer with no heterologous peptide sequence added, so it is directly fused without a linker and formulated with an adjuvant to enhance the magnitude, breadth, and durability of the immune response. Other authors have disclosed homodimers with interesting results^11,12^, but PHH-1V is based on a fusion heterodimer consisting of SARS-CoV-2 variants other than the ancestral strain assessed as primary series.

Pre-clinical studies performed in mice, pigs, and non-human primates have shown that this vaccine candidate is safe and immunogenic, inducing a high titer of neutralizing antibodies against all the studied variants of concern (VOCs) of SARS-CoV-2, and promoting the activation of CD4+ and CD8 + T lymphocytes with a balanced Th1/Th2 response. Moreover, the PHH-1V vaccine has demonstrated to be efficacious against an experimental infection with SARS-CoV-2 in K18-hACE2 mice and cynomolgus monkeys^13,14^.

In Catalonia, Spain, we conducted a first-in-human phase 1-2a clinical trial in healthy, SARS-CoV-2 seronegative adults, younger than 40 years old, to evaluate the safety, reactogenicity and immunogenicity of 10 µg, 20 µg and 40 µg doses of PHH-1V. Here we report our findings.

## Results

### Trial population

**Fig. 1:**
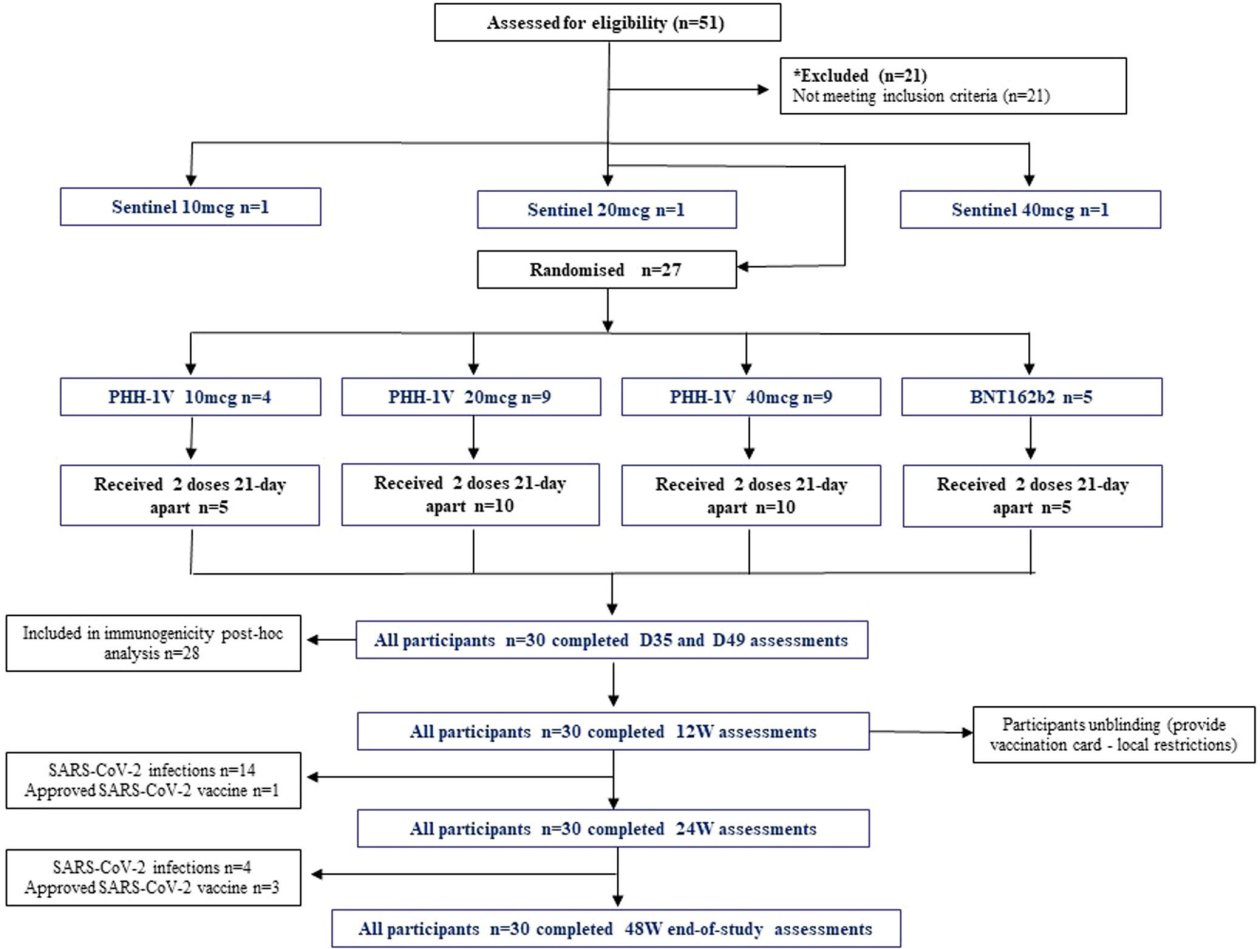
Subject disposition thorough the study duration according to Consort flow diagram.

Between August 16 and September 2, 2021, 51 healthy adults were screened and after the eligibility assessment 21 were excluded. Of the 30 participants included, three were allocated as sentinels at each PHH-1V dose group and the remaining 27 were randomly assigned into four groups to receive PHH-1V 10 µg (*n* = 4), PHH-1V 20 µg (*n* = 9), PHH-1V 40 µg (*n* = 9) or control vaccine BNT162b2 (*n* = 5). Thirteen participants were female (43.3%), the mean age was 27.7 (SD 4.91) and most of them were Hispanic (*n* = 29, 96.7%). All participants completed the 2-dose scheme and attended all study visits as scheduled. Subject disposition is shown in Fig. 1. Due to the restrictions imposed to the Catalan population during the 6th COVID-19 wave and, in an intent to minimize the interference in daily-life activities of the study participants, in December 2021, coinciding with the 12 weeks visit, the study was single-unblinded and a non-travel Catalan vaccination certificate was issued for participants allocated to study vaccine and a standard Spanish vaccination certificate for the control group. Participants signed a confidentiality agreement not to disclose the allocation group to investigators who were to continue the vaccine safety evaluation. Participants were informed about safety and immunogenicity results obtained so far in the study and a safety-oriented recommendation was given as for receiving any available approved COVID-19 vaccine. All individuals were asked to continue their participation in the study until completion. All these changes were approved by the Spanish Agency of Medicines and Medical Products (AEMPS) and the Research Ethics Committee (REC). The trial follow-up was completed in September 2022.

### Safety and reactogenicity

**Fig. 2:**
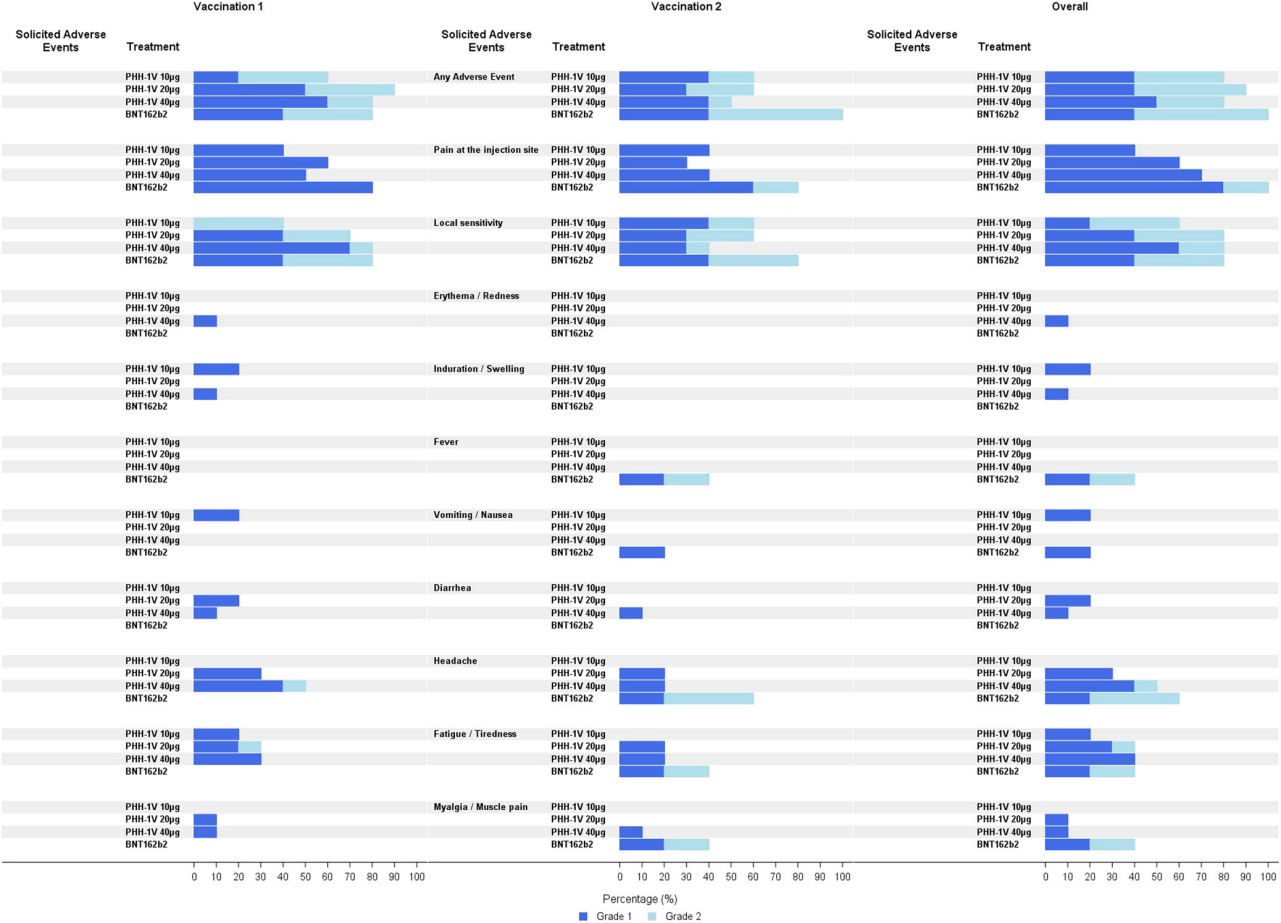
Local and Systemic Solicited Adverse Events (AE) within 7 days after 1 and 2 doses of study vaccines. Solicited AE are reported by percentage. Percentage of Solicited AE grade 1 are shown in dark blue bars and grade 2 are shown in light blue bars. These AE were reported by participants in their diaries as well as during follow-up visits.

Vaccines were safe and well tolerated. All solicited adverse events (AE) reported were mild to moderate (grade 1 and 2), transient and resolved within the reporting period. Twenty-six subjects (86.7%; 95%IC: 69.3%–96.2%) referred to having at least one solicited AE within 7 days after the first or the second vaccine dose; 24 (80.0%; 95%IC: 61.4%–92.3%) after the first vaccination and 19 (63.3%; (95%CI: 43.9%–80.1%) after the second one. The most common solicited events for all groups were tenderness and pain at the site of injection followed by headache and fatigue (Fig. 2). Two participants from the BNT162b2 control group had fever, defined as temperature ≥38 °C, within 7 days after the second vaccination. No prophylactic treatment was prescribed. Eight participants, four in PHH-1V 20 µg, one in PHH-1V 40 µg and three in the control group, took occasional paracetamol and other painkillers within 7 days after vaccination.

From day 0 through day 28 following vaccinations, 25 (83.3%) participants reported 61 mild or moderate unsolicited AE, and 35 were considered related to the vaccines (PHH-1V 10 µg *n* = 8, 20 µg *n* = 13, 40 µg *n* = 12, BNT162b2 *n* = 2). The most frequent unsolicited events described were related to the respiratory tract (*n* = 14); in all these cases a SARS-CoV-2 polymerase chain reaction (PCR) test was performed, and COVID-19 infection was discarded. Within this assessment period, there were four medial attended AE (MAAE) reported (for 3 subjects): vulvovaginitis and acute asthma exacerbation considered related to study vaccines; bronchitis and common cold reported as unrelated. The distribution of these MAAE by study vaccine was: three in the PHH-1V 20 µg group and one in the PHH-1V 40 µg group. In addition, there was a laboratory abnormality finding, a grade 3 hypoglycemia, completely asymptomatic and resolved.

All participants reported at least 1 unsolicited AE thorough the duration of the study, the event characteristics and distribution by vaccine group are shown in Table 1. No AE of special interest (AESI) were reported.

**Table 1:** Number of unsolicited adverse events reported thorough the study duration according to their severity, if medically attended and their relation to the study vaccines according to vaccine cohort.

	RELATED				UNRELATED				
VACCINES GROUPS									
	PHH-1V 10 µg	PHH-1V 20 µg	PHH-1V 40 µg	BNT162B2	PHH-1V 10 µg	PHH-1V 20 µg	PHH-1V 40 µg	BNT162B2	TOTAL
GRADE 1–2	8	14	12	2	14	35	24	9	118
GRADE 3–4^a^	0	0	0	0	0	3	0	2	5
TOTAL	8	14	12	2	14	38	24	11	123
*MAAE*^*b*^	*0*	*1*	*1*	*0*	*4*	*12*	*5*	*5*	*28*

	PHH-1V 10 µg	PHH-1V 20 µg	PHH-1V 40 µg	BNT162B2
COVID-19 cases	2	7	5	5
Asymptomatic	0	0	2	0
Other vaccines	2	0	1	1

Number of participants having an asymptomatic SARS-CoV-2 infection or mild to moderate COVID-19 and participants receiving at least one dose of approved vaccines according to vaccine cohort.

^a^Grade 3–4 events reported were laboratory abnormality findings: 4 episodes of hypoglycemia and 1 episode of hyperkalemia, all transient and asymptomatic.

^b^MAAE: medically attended adverse events, these events are those unsolicited adverse events grade 1–4 that required medical attention. Most unsolicited adverse events reported were related to the respiratory tract such as nasopharyngitis, bronchitis, cough, and rhinorrhea.

There were 2 severe AE (SAE) reported from 1 participant in the PHH-1V 40 µg: acute appendicitis and intestinal adhesions, both happened after 28 days from the second vaccine dose, were considered unrelated to the study vaccine and resolved.

After 12 weeks from second vaccination (12 W), 18 participants had a SARS-CoV-2 infection, a few asymptomatic and the rest reported as mild, and all recovered. SARS-CoV-2 infection characteristics and distribution by vaccine group are shown in Table 1. Four study participants received at least one dose of an approved mRNA vaccine between the 12 W and 48 weeks visits, of these, two have had a SARS-CoV-2 infection 6 months before.

### Immunogenicity

**Fig. 3:**
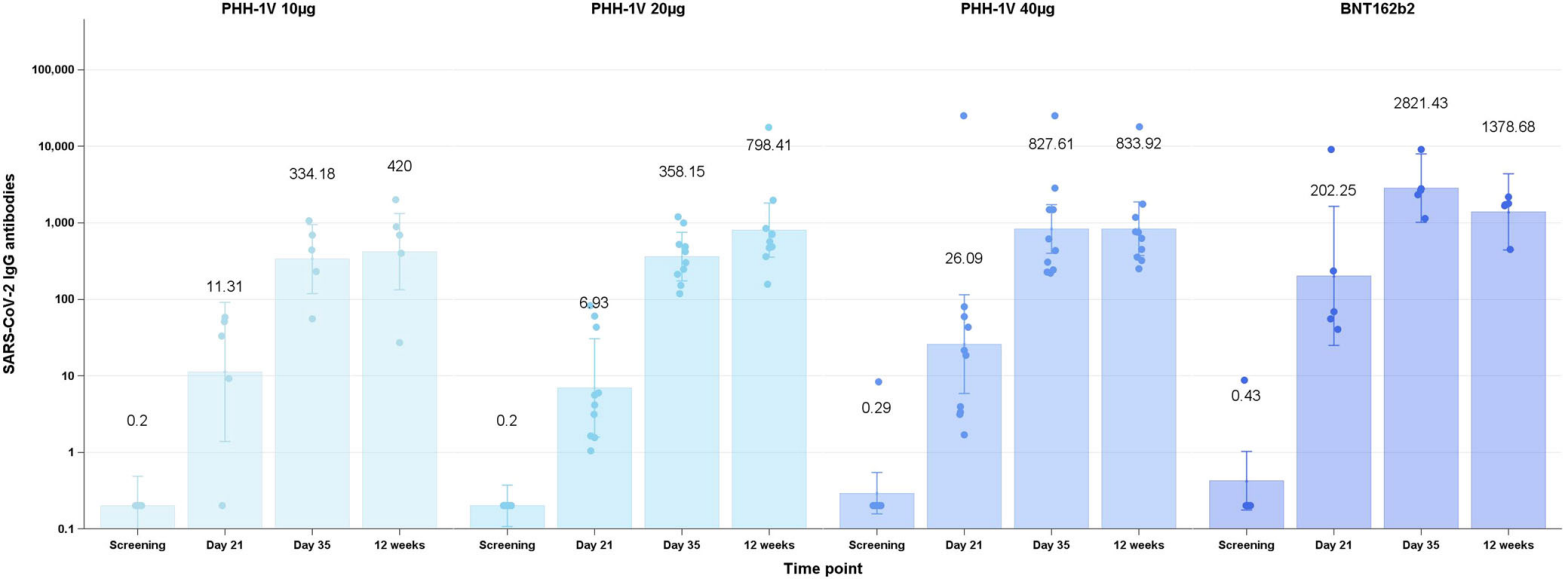
Total binding antibodies after 1 and 2 doses of study vaccines. Total binding antibodies at screening, day 21 and day 35 after the first vaccination and 12 weeks after receiving the second dose are shown for all participants distributed by vaccine group PHH-1V 10 µg *n* = 5, PHH-1V 20 µg *n* = 10, Cohort 3 PHH-1V 40 µg *n* = 10, control *n* = 5. Binding is expressed as geometric mean titers (GMT). Bars indicate 95% confidence intervals and data points represent individual titers.

Total binding antibodies titers were assessed by enzyme-linked immunosorbent assay (ELISA). At baseline, all participants but two had binding antibodies titers lower than the limit of detection (0.8 U/ml), one female in the PHH-1V 40 µg group (8.4 U/ml) and another female in the control group (8.77 U/ml). By day 21, twenty-nine (97%) participants had seroconverted, and binding antibodies had >4-fold change from baseline. The only participant that had undetectable levels had received a dose of PHH-1V 10 µg. At day 35, 14 days after second vaccination, all participants had seroconverted and 100% had >4-fold change. Binding antibodies geometric mean titers (GMT) at screening, day 21 and day 35 after first vaccination and 12 weeks after second vaccination can be seen in Fig. 3. Geometric mean fold rise (GMFR) between baseline and relevant timepoints for each vaccine group was as follows: PHH-1V 10 µg: (1) day 21 [56.56 (95% CI 11.44–279.65); *p* < 0.0001], (2) day 35 [1670.88 (95% CI 708.99–3937.80); *p* < 0.0001], (3) 12 weeks [2100.02 (95% CI 789.14–5588.49); *p* < 0.0001]; PHH-1V 20 µg: (1) day 21 [34.64 (95% CI11.19–107.24); *p* < 0.0001], (2) day 35 [1790.73 (95% CI 976.72–3283.17); *p* < 0.0001], (3) 12 weeks [2687.92 (95% CI 1332.36–5422.64); *p* < 0.0001]; PHH-1V 40 µg: (1) day 21 [89.77 (95% CI 29–277.94); *p* < 0.0001], (2) day 35 [2847.56 (95% CI 1553.14–5220.77); *p* < 0.0001], (3) 12 weeks [2869.24 (95% CI 1436.14–5732.42); *p* < 0.0001]; control vaccine: (1) day 2 [1474.75 (95% CI 96.02–2347.35); *p* < 0.0001], (2) day 35 [6622.85 (95% CI 2810.2–15608.22); *p* < 0.0001], (3) 12 weeks [3236.23 (95% CI 1216.1–8612.12); *p* < 0.0001].

**Fig. 4:**
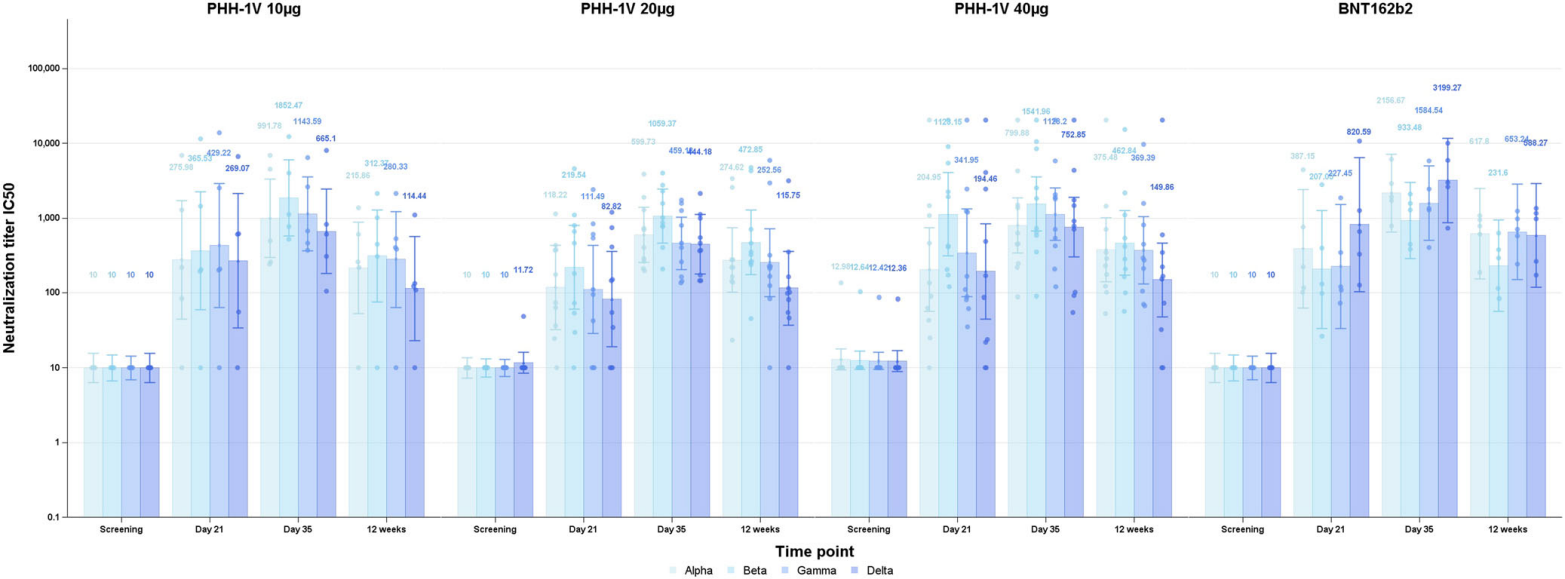
Neutralizing responses after 1 and 2 doses of study vaccines by pseudo-virus neutralization assay (PNBA). Neutralization is expressed in geometric mean titers (GMT). The 50% neutralization titers against alpha (B.1.1.7), beta (B.1.351), delta (B.1.617.2) and gamma (P.1) variants of concern (VOC) assessed are shown at screening, 21 and 35 days after the first vaccination and 12 weeks after the second vaccination for all participants distributed by vaccine group PHH-1V 10 µg *n* = 5, PHH-1V 20 µg *n* = 10, Cohort 3 PHH-1V 40 µg *n* = 10, control *n* = 5. Bars indicate 95% confidence intervals and data points represent individual 50% neutralization titers.

Neutralization assays were conducted using replicative SARS-CoV-2 isolates or pseudoviruses. The correlation between these two techniques was excellent for the alpha variant (*r* = 0.93, *p* < 0.0001). The pseudovirus-based neutralization assay (PBNA) analyzed VOCs present at that moment (alpha, beta, gamma and delta). The participant in the PHH-1V 40 µg group with detectable binding antibodies at baseline also had detectable neutralizing antibodies against all VOCs by PBNA at the same time-point; this was interpreted as a cross-reaction to other coronaviruses or a previous SARS-CoV-2 asymptomatic infection with a false-negative IgG antibody detection at the screening. On day 21, twenty-nine participants had detectable titers of neutralizing antibodies by PBNA to all the studied VOCs as follows: PHH-1V 10 µg *n* = 4, PHH-1V 20 µg *n* = 10, PHH-1V 40 µg *n* = 10, BNT162b2 *n* = 5. All participants had detectable neutralizing antibodies for all VOCs at day 35. Neutralization GMT for all VOCs at screening, day 21, day 35 and 12 weeks can be seen in Fig. 4 and Table 2. GMFR for relevant timepoints are shown in Table 2. Similar results were obtained when a full replicative virus neutralization assay was carried out (data not shown). Considering that the two participants that had baseline antibody titers above the limit of detection could have had a previous SARS-CoV-2 infection, we also conducted a post-hoc analysis excluding them. This additional analysis did not modify the main conclusions of the study (data not shown).

**Table 2:** Neutralization antibodies titres measured by pseudovirus-based neutralization assay (PBNA) by vaccine group, variant of concern assessed and time-points: screening, 21 and 35 days after first vaccination and 12 weeks after second vaccination.

	PHH-1V 10 µg (*n* = 5)	PHH-1V 20 µg (*n* = 10)	PHH-1V 40 µg (*n* = 10)	BNT162b2 (*n* = 5)
ALPHA B.1.1.7				
Screening^1^	10 (6.4–15.63)	10 (7.29–13.71)	12.98 (9.47–17.8)	10 (1.24) (6.4–15.63)
Day 21	275.98 (44.72–1703.03)	118.22 (32.65–428.11)	204.95 (56.6–742.17)	387.15 (62.74–2389.01)
Day 35^1^	991.78 (298.31–3297.38)	599.73 (256.46–1402.47)	799.88 (342.0.5–1870.51)	215.66 (648.68–7170.27)
12 Week^1^	215.86 (56.94–818.29)	190.69 (73.25–496.42)	375.48 (146.34–963.43)	617.8 (162.97–2341.99)
Day 35 vs screen^2^	99.18 (34.87–282.06); *p* < 0.0001	59.97 (28.64–125.58); *p* < 0.0001	61.61 (29.42–129.01); *p* < 0.0001	215.67 (75.83–613.34); *p* < 0.0001
12 Week vs screen^2^	21.59 (6.91–67.4); *p* < 0.0001	19.07 (8.38–43.39); *p* < 0.0001	28.92 (12.93–64.7); *p* < 0.0001	61.8 (19.78–192.92); *p* < 0.0001
BETA B.1.351				
Screening^1^	10 (6.7–14.93)	10 (7.53–13.27)	12.64 (9.52–16.78)	10 (6.7–14.93)
Day 21	365.53 (59.66–2239.72)	219.54 (60.93–791.04)	1123.14 (311.71–4046.89)	207.05 (33.79–1268.67)
Day 35^1^	1852.47 (572.79–5991.17)	1059.37 (461.95–2429.42)	1541.96 (672.39–3536.13)	933.47 (288.63–3018.99)
12 Week^1^	312.37 (79.99–1219.8)	350.09 (130.44–939.63)	462.84 (176.64–1212.73)	231.6 (59.31–904.4)
Day 35 vs screen^2^	185.25 (63.56–539.95); *p* < 0.0001	105.94 (49.72–225.72); *p* < 0.0001	122 (57.26–259.95); *p* < 0.0001	93.35 (32.03–272.08); *p* < 0.0001
12 Week vs screen^2^	31.24([9.24–105.58) *p* < 0.0001	35.01 (14.41–85.08); *p* < 0.0001	36.62 (15.48–86.64); *p* < 0.0001	23.16 (6.85–78.28); *p* < 0.0001
DELTA B.1.617.1				
Screening^1^	10 (6.36–15.72)	11.72 (8.51–16.14)	12.36 (8.97–17.02)	10 (6.36–15.72)
Day 21	269.07 (34.1–2122.97)	82.81 (19.22–356.82)	194.46 (45.13–837.85)	820.59 (104–6474.58)
Day 35^1^	665.1 (181.02–2443.73)	444.17 (176.98–1114.77)	752.85 (299.97–1889.49)	3199.27 (870.73–11754.85)
12 Week^1^	114.44 (25.78–508.08)	72.5 (24.9–211.05)	149.86 (52.23–429.97)	588.27 (132.5–2611.78)
Day 35 vs screen^2^	66.51 (20.18–219.26); *p* < 0.0001	37.89 (16.3–88.08); *p* < 0.0001	60.93 (26.21–141.62); *p* < 0.0001	319.93 (97.05–1054.68); *p* < 0.0001
12 Week vs screen^2^	11.44 (3.15–41.62); *p* = 0.0006	6.18 (2.44–15.67); *p* = 0.0004	12.13 (4.87–30.22); *p* < 0.0001	58.83 (16.18–213.93); *p* < 0.0001
GAMMA P.1				
Screening^1^	10 (6.91–14.48)	10 (7.7–12.99)	12.42 (9.56–16.13)	10 (6.91–14.48)
Day 21	429.22 (63.72–2891.43)	111.49 (28.94–429.58)	341.95 (88.75–1317.5)	227.45 (33.76–1532.18)
Day 35^1^	1143.59 (364.67–3586.22)	459.18 (204.65–1030.32)	1128.2 (502.81–2531.45)	1584.54 (505.29–4969.02)
12 Week^1^	280.33 (70.98–1107.23)	158.99 (58.79–429.96)	369.39 (139.85–975.7)	653.24 (165.39–2580.1)
Day 35 vs screen^2^	114.36 (41.34–316.38); *p* < 0.0001	45.92 (22.36–94.29); *p* < 0.0001	90.87 (44.25–186.61); *p* < 0.0001	158.45 (57.27–438.38); *p* < 0.0001
12 Week vs screen^2^	28.03 (8.03–97.88); *p* < 0.0001	15.9 (6.4–39.49); *p* < 0.0001	29.75 (12.29–72.03); *p* < 0.0001	65.32 (18.71–228.07); *p* < 0.0001

*GMT* geometric mean titer, *CI* confidence interval, *GMFR* geometric mean fold rise.

^1^Neutralization titers measured as IC_50_ are expressed in GMT shown as adjusted treatment mean (95% CI).

^2^GMFR is shown as fold rise of adjusted treatment means between time-points and screening (CI 95%).

T-cell mediated immunogenicity was assessed using interferon gamma enzyme-linked immunosorbent spot (IFN-γ ELISPOT). At day 35 after first vaccination results showed that vaccination with PHH-1V 40 µg induced a specific T-cell response, with a significant IFN-ɣ production after re-stimulation in vitro with RBD peptides from alpha [2.07 SFC/106 PBMC (95% CI 1.03–4.17) *p* = 0.0417] and delta [2.99 SFC (95% CI 1.2–7.45) *p* = 0.0208] SARS-CoV-2 variants compared with baseline and this effect was maintained 12 weeks after second vaccination. There was a trend for the other RDB variants analyzed but not statistically significant. Changes in the T-cell responses between baseline and relevant time-points for all VOCs assessed can be seen in Table 3. Supplementary fig. 1 provides an overview of T-cell mediated responses to VOCs assessed at relevant timepoints by vaccine group. We had technical problems when analyzing samples by intracellular cytokine staining (ICS), thus we exclude it from our report.

**Table 3:** Changes in SARS-CoV-2 specific T-cell responses to variants of concern assessed between relevant timepoints by vaccine group as measured by IFN-γ ELISPOT.

	PHH-1V 10 µg (*n* = 5)	PHH-1V 20 µg (*n* = 10)	PHH-1V 40 µg (*n* = 10)	BNT162b2 (*n* = 5)
RBD ALPHA B.1.1.7				
Day 21 vs screening	0.29 (0.12–0.71); *p* = 0.0085	0.7 (0.37–1.32); *p* = 0.2594	2.49 (1.32–4.67); *p* = 0.0063	2.5 (0.97–6.46); *p* = 0.0573
Day 35 vs screening	0.65 (0.24–1.74); *p* = 0.3763	0.91 (0.45–1.82); *p* = 0.7755	2.07 (1.03–4.17); *p* = 0.0417	8.2 (2.9–23.21); *p* = 0.0003
12-week vs screening	1.5 (0.26–8.8); *p* = 0.6423	2.1 (0.63–7.03); *p* = 0.2167	3.71 (1.06–13.05); *p* = 0.0413	1.5 (0.26–8.8); *p* = 0.6423
RBD BETA B.1.351				
Day 21 vs screening	0.52 (0.25–1.1); *p* = 0.0831	0.54 (0.32–0.91); *p* = 0.0221	2.17 (1.28–3.67); *p* = 0.0058	6.05 (2.7–13.55); *p* = 0.0001
Day 35 vs screening	0.66 (0.2–2.18); *p* = 0.4774	1.35 (0.58–3.17); *p* = 0.4698	1.24 (0.53–2.91); *p* = 0.6022	16.28 (4.7–56.35); *p* < 0.0001
12-week vs screening	2.98 (0.58–15.23); *p* = 0.1813	1.85 (0.58–5.9); *p* = 0.2889	1.86 (0.55–6.35); *p* = 0.3062	27.43 (4.7–160.11); *p* = 0.0007
RBD DELTA B.1.617.1				
Day 21 vs screening	0.43 (0.17–1.11); *p* = 0.0801	0.85 (0.43–1.66); *p* = 0.6134	1.67 (0.85–3.27); *p* = 0.1304	5.94 (2.2–16.04); *p* = 0.0011
Day 35 vs screening	1.05 (0.29–3.82); *p* = 0.9414	1.57 (0.63–3.91); *p* = 0.3210	2.99 (1.2–7.45); *p* = 0.0208	19.31 (5.14–72.6); *p* < 0.0001
12-week vs screening	1.16 (0.25–5.42); *p* = 0.8492	2.25 (0.77–6.59); *p* = 0.1335	3.29 (1.07–10.04); *p* = 0.0379	15.79 (3.18–78.49); *p* = 0.0015
RBD PEPTIDE MIX				
Day 21 vs screening	0.34 (0.1–1.16); *p* = 0.0831	0.63 (0.27–1.49); *p* = 0.2845	1.16 (0.49–2.74); *p* = 0.7257	3.16 (0.9–11.17); *p* = 0.0718
Day 35 vs screening	0.94 (0.31–2.82); *p* = 0.9083	1.18 (0.54–2.58); *p* = 0.6588	1.67 (0.77–3.63); *p* = 0.1882	11.01 (3.48–34.82); *p* = 0.0002
12-week vs screening	15.87 (2.61–96.36); *p* = 0.0041	0.71 (0.11–4.52); *p* = 0.7055	2.37 (0.66–8.51); *p* = 0.1768	1.67 (0.45–6.18); *p* = 0.4303

T-cell response is expressed as mean spot forming cells/106 PBMC (95% CI).

*RBD* receptor binding domain, *CI* confidence interval.

Within 12 W and 24 W visits 50% participants, and at the end of the study (48 weeks) almost 70% (*n* = 20) have had a SARS-CoV-2 infection or had been vaccinated with an approved vaccine. Humoral and cellular immunogenicity data affected was censored and as can been seen in Supplementary table 1. However, we consider that is not possible to draw any reliable conclusion with the resulting small sample size and different antigen exposures by vaccination and/or infection.

## Discussion

Vaccination remains an essential component of the approach to fighting against the ongoing pandemic, but limited supply, storage requirements and vaccine hesitancy have restricted their global impact. As we face the rapid emergence and spread of new variants, vaccines keep losing their efficacy, so developing vaccines with broad neutralizing activity against variants of SARS-CoV-2 which provide cross-strain protection seems the recommended strategy to pursue. Adjuvanted protein-based vaccines, using a more traditional inexpensive technology and already in widespread use for other diseases, have a lot to offer and could help to change the pandemic^15^.

PHH-1V vaccine has shown to be safe and well-tolerated in healthy young adults. Furthermore, the two-dose regimen induced a robust antibody response, with both binding and neutralizing activities, against all the different VOCs assessed. Responses were maintained 12 weeks after the second dose, although, as could be expected, with a decreasing trend. In addition, PHH-1V vaccine elicited a moderate specific T-cell response at the highest dose of 40 µg.

PHH-1V vaccine safety profile is comparable to other vaccines using the same platform technologies^16^. There were no severe adverse events related to the study vaccine or study withdrawals. The most frequent AE were local pain and tenderness and most of them reported after the first dose of PHH-1V vaccine contrarily to the control group in which most of solicited AE were reported after the second dose, as has already been described^17^. Fever was only described in the control group and although no prophylactic antipyretic was prescribed, proportionally more participants in the control group had to take occasional paracetamol or non-steroidal painkillers after vaccinations. Overall, PHH-1V has a good safety profile, thus we have continued this evaluation at a larger and international scale without any safety concerns. Interesting to point out that participants as well as investigators were blinded during the main safety assessments, which had somehow limited the bias. At the end of 2021, after 12 weeks from the second vaccine dose administration, there was another SARS-CoV-2 peak in Spain, coinciding with the Omicron VOC detection^18^ and soon began branching off into several subvariants. This variant and its descendent lineages have since dominated the variant landscape. Also, during this peak, 50% of the participants in our study were infected by SARS-CoV-2. COVID-19 cases in our study, contrary to those previously reported since the beginning of the pandemic, were mostly mild with only upper respiratory symptoms, as has been described for omicron breakthrough-infected individuals, particularly in those vaccinated^19^.

All study participants had seroconverted after 14 days from the second dose and at that same time point, they all had a >4-fold increase in neutralizing antibodies titers. It is presumed that neutralizing antibodies are associated with protection^9,20^. PHH-1V 10 µg was fairly immunogenic but at higher doses there was a robust increase in neutralizing activity, yet evident after the first dose and significantly enhanced after the second, similar as to what has been shown by other SARS-CoV-2 protein vaccines^21,22^. In the different clinical trials evaluating the efficacy of SARS-CoV-2 vaccines that nowadays are widely used, neutralization GMT were assessed and are also considered as surrogate markers of protection. Unfortunately, different analytical methods were used, and the results vary for the different vaccines making indirect comparison between vaccines not reliable^23,24^. Nevertheless, research groups continue the task of finding predictive models of immune protection with encouraging results^9,25^. Taking all this into account, we could consider that PHH-1V neutralization response may be associated with a protective effect against SARS-CoV-2 infection.

We did not observe a clear increase of specific T-cell responses against all studied RBD VOCs after exposing PBMC from vaccinated individuals to peptide pools. This could be due to different reasons. First, the limited number of participants per group did not allow us to assess sufficient reliable immunogenicity data to achieve statistical significance, even if most T-cell epitopes have been reported to be conserved at the sequence level across the different variants^26,27^. Furthermore, and although not analyzed, it is conceivable to suggest that at least specific CD4 + T-cells were clearly induced in order to stimulate a robust humoral response^28^. Moreover, since PHH-1V is a protein-based vaccine, we expected lower specific T-cell frequencies than with other vaccine platforms, as previously described^29^.

Based on the safety and immunogenicity data obtained in this study, the two-dose regimen of 40 µg PHH-1V was determined to be the optimal dose scheme to continue with the development plan of the PHH-1V vaccine, however due to the evolution of the pandemic and successful vaccine coverage in Europe with first generation vaccines, PHH-1V has been further evaluated as a booster^30^ with sufficiently robust data on the quality, safety, and immunogenicity that the EMA´s human medicines committee (CHMP) has recently recommended its marketing authorization in the European Union^31^.

This study has limitations. The interpretation of the results is limited due to the nature of the study itself, which was designed for dose-selection and initial safety assessment with a small sample size. This initial safety assessment could not be finalized unbiased, since we had to unblind the study for participants, in an attempt to life-balance with all the restrictions imposed then, such as the requirement of vaccination or infection-recovery certificate to enter a restaurant. Furthermore, most of our participants had a SARS-CoV-2 infection before the study ended, thus longer-term immunogenicity assessment was compromised. Another limitation is the lack of immunogenicity data for the Omicron VOC or any of its subvariants, however it has been assessed in all other studies within the clinical development with good results. All the participants were very young, therefore, safety, tolerability and immunogenicity results obtained in this study cannot be extrapolated to other populations such as children, elderly or immunocompromised.

In conclusion, we found that this recombinant protein RBD fusion heterodimer vaccine PHH-1V is safe, well tolerated, and immunogenic in healthy, young people as primary series. This initial evaluation has significantly contributed to its further development. PHH-1V is already part of the European vaccine portfolio and is expected to be a valuable addition to the global COVID-19 response.

## Methods

### Study design and participants

This was a first-in-human phase 1-2a dose-escalation, randomized, double-blinded, active-comparator controlled clinical trial conducted at two centers in Catalonia, Spain (Hospital Clínic de Barcelona in Barcelona city and Hospital Universitari Dr Josep Trueta in Girona city) to evaluate safety and immunogenicity of a recombinant protein RBD fusion heterodimer vaccine against SARS-CoV-2. Trial screenings started on August 16, 2021, and the study follow-up was completed 48 weeks after the last vaccine dose. The HIPRA-HH-1 study was approved by the Spanish Agency of Medicines and Medical Products (AEMPS) and the Research Ethics Committee (REC) of the Hospital Clínic de Barcelona and was overseen by an independent Data Safety Monitoring Board (DSMB).

Eligible participants were healthy women and men adults 18–39 years of age, willing to avoid receiving any other vaccine within 4 weeks before and after each vaccine dose administered in the trial and with a body mass index between 18 and 40 kg/m^2^ at screening. The age range limitation was recommended by the REC given that, when the study was evaluated, other approved COVID-19 vaccines were easily available in Catalonia for anyone ≥40 years old. Participants were recruited through advertising on the site’s website and social media. Subject recruitment material was reviewed and approved by the REC. At screening, all volunteers were tested for IgG antibodies against SARS-CoV-2 as evidence of previous infection and a polymerase chain reaction (PCR) test was used to assess acute infections. Participants with any positive test were excluded along with participants recently exposed to persons with confirmed SARS-CoV-2 infection. Female participants of childbearing potential and men had to agree to use highly effective methods of contraception. The participant’s medical history was assessed by the study investigators in addition to reviewing clinical and laboratory findings from tests at screening following the study protocol. All participants provided written informed consent before enrollment in the trial.

### Randomization and masking

Participants were allocated to the 3-dose escalation cohorts according to the order that they had been preselected considering the laboratory results and subject availability. The cohorts were composed as follows: Cohort 1 PHH-1V 10 µg *n* = 5, control vaccine *n* = 1; Cohort 2: PHH-1V 20 µg *n* = 10, control vaccine *n* = 2; Cohort 3 PHH-1V 40 µg *n* = 10, control vaccine *n* = 2. Each cohort had a safety sentinel individual that received the study vaccine of the corresponding dose. Except for sentinels, all participants at each dose cohort were randomly allocated to study vaccine or control vaccine in a 5:1 allocation scheme. A centralized computer-generated randomization was used, and a study independent statistician generated these randomization codes by means of the PROC PLAN of the SAS® system. Randomization was centralized through the electronic Case Report Form (eCRF) created using the Elsevier Macro® system. This system is regulatory compliant (ICH GCP and FDA 21 CFR Part11). Study investigators and participants were both blinded, only study staff responsible for preparing and administering the vaccine were unblinded and were not involved in assessment of study data. Syringes were masked using opaque labels since study and control vaccines were visually different.

### Study vaccine

Initially, the study vaccine was based on sequence of the SARS-CoV-2 strain first detected in Wuhan, but due to the rapid spread of new Variants of Concern (VOCs) around the world, the sponsor decided to develop a new antigen candidate, based on the same Chinese Hamster Ovary (CHO) cells platform technology, considering variants B.1.351 and B.1.1.7. This new candidate also elicited a cross-reactive response and neutralization against heterologous pseudoviruses Wuhan strain, P.1 and B.1.617.2 in pre-clinical studies^14^. Different adjuvants, alone or combined, were assayed. Based on non-clinical studies, the oil-in-water emulsion adjuvant SQBA was selected. Study vaccines were packed as single vials with 0.5 ml emulsion ready to use and were stored at 2–8 °C. Due to AEMPS and REC recommendations we included a comparator control group, only considering safety assessment. This comparator group was an approved mRNA vaccine, BNT162b2, and its selection was made considering the similar posology^32^. The study vaccine was developed and provided by HIPRA (Amer, Girona, Spain) and as Sponsor, was involved in the trial design, data collection, data analysis, data interpretation, contributed to drafting the manuscript and other activities pertaining to its role as defined by the International Council for Harmonization (ICH) E6(R2) guideline for Good Clinical Practice^33^.

### Study endpoints

**Fig. 5:**
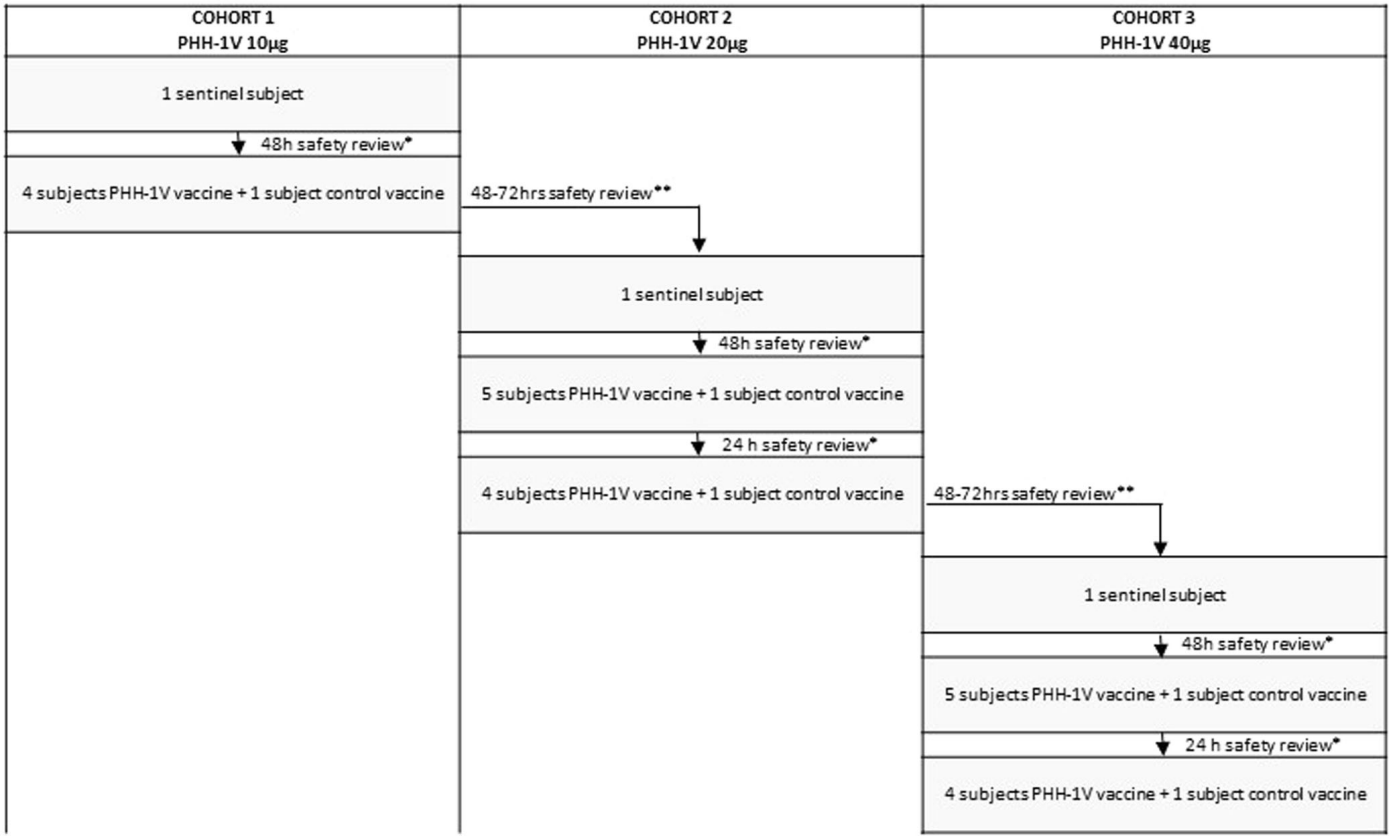
Dose-selection flow-chart. Sentinel subjects and subsequent 6 participants in each cohort (5 in the first cohort) will be closely observed on site during 2 h after the first dose. All other participants and all second vaccinations will be monitored on site during 1 h after vaccination. *Assessments by the IRC review (Internal Review Committee). **Assessments by the DSMB review (Data and Safety Monitoring Board).

The primary endpoint was safety and tolerability of study vaccine, and it was assessed as solicited local and systemic reactogenicity adverse events within 7 days following each vaccination and unsolicited local and systemic reactogenicity adverse events within 28 days following each vaccination. Changes in safety laboratory parameters at 7 days following each vaccination were assessed as a secondary safety endpoint. Serious Adverse Events (SAE), Adverse Events of Special Interest (AESI) and Medically Attended Adverse Events (MAAE) were monitored throughout the study duration. Secondary endpoints related to immunogenicity were defined as antibody neutralization measured as IC50 or ID50 and expressed as Geometric Mean Titers (GMT) and Geometric Mean Fold Rise (GMFR) from baseline to 21 days and 35 days after first vaccination and 12 weeks (12 W), 24 weeks (24 W), and 48 weeks (48 W) after the second vaccine dose; binding antibodies titer measured as GMT and GMFR from baseline to 21 days and 35 days after first vaccination and 12 W, 24 W and 48 W after the second vaccine dose; and T-cell mediated response measured by an Interferon gamma Enzyme-Linked Immunosorbent Spot assay (IFN-γ ELISpot) and Intracellular Cytokine Staining (ICS) at baseline and 35 days after first vaccination. See study timeline in Fig. 5. Exploratory immunogenicity endpoints were T-cell mediated response by ELISpot 21 days after first vaccination and 12 W and 24 W after the second vaccine dose; and seroconversion. Seroconversion was defined in two ways: as ≥4-fold change in binding antibody titer and a titer above 0.8 U/mL from baseline to 21 days and 35 days after the first vaccination. Other exploratory endpoints related to immunogenicity and to COVID-19 cases were assessed.

### Study interventions

**Fig. 6:**
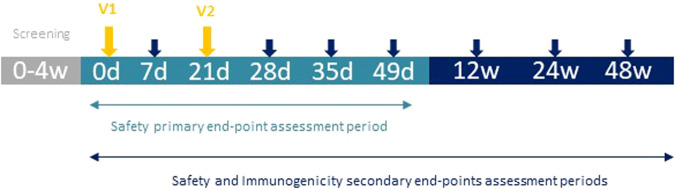
Study design: timeline and interventions. In gray the screening period, in light blue the primary safety endpoint assessment period, arrows in yellow represent vaccine administration; in dark blue the secondary endpoint assessment period and arrows in dark blue represent blood extractions for safety and immunogenicity assessments.

All vaccines were administered as a single intramuscular injection into the deltoid muscle at days 0 and 21. Each sentinel individual in each dose cohort was monitored by phone for 24 and 48 h after the first administration. Early safety data from sentinels was reviewed by an Internal Review Committee (IRC) before including the remaining participants of each group. Further participants in the same cohort were randomized to receive either study vaccine or control vaccine and were distributed in small groups of five-to-six participants per day and safety data was monitored for 24 h. After 48–72 h of the last vaccine administered in each dose cohort, DSMB assessed if any clinically significant adverse events occurred and if no halting rules were met, study vaccine dose was escalated. All participants were observed for at least 60 min after each vaccine dose on site. Participants from the same cohort received the vaccine with an interval of 60 min between them. See dose escalation flow chart in Fig. 6.

During the first 7 days after each vaccination, any solicited local and systemic AE were self-reported by participants daily on the diary cards and verified by the investigator during the scheduled visit. Solicited local AE included pain, tenderness, erythema, and swelling; and solicited systemic AE included fever, nausea/vomiting, diarrhoea, headache, fatigue, and myalgia. Unsolicited local and systemic AE occurring within the 28 days after each vaccination were reported by participants during scheduled follow-up visits or by any preferred method if occurring before. All unsolicited AE, SAE, AESI or MAAE were monitored throughout the study duration. Laboratory safety tests including routine blood and serum chemistry were done to assess any short- and long-term toxicity after vaccination. AE and changes in laboratory tests were assessed according to the Guidance for Industry, Toxicity Grading Scale for Healthy Adult and Adolescent Volunteers Enrolled in Preventive Vaccine Clinical Trials by the U.S. Department of Health and Human Services, FDA, Center for Biologics Evaluation and Research (September 2007). Blood samples for safety assessment were collected at screening, 7 and 28 days after each vaccine dose and at 12 W, 24 W and 48 W after the second vaccine dose. To assess the immunogenicity, blood samples were collected at screening, 21 and 35 days after the first vaccine dose and 12 W, 24 W and 48 W after the second vaccination. See study timeline in Fig. 2.

### Immunogenicity assessments

#### Binding antibodies

The in vitro quantitative determination of binding antibodies (including IgG) against RBD was assessed by the percentage of subjects having a ≥4-fold increase in the binding antibodies using the Elecsys SARS-CoV-2 immunoassay (Roche Diagnostics GmbH, D-68305 Manheim). The assay used a recombinant protein representing the RBD in a double-antigen sandwich assay format, which favors detection of high affinity antibodies against this SARS-CoV-2 and expressed as the GMT.

#### Neutralizing antibodies

The neutralization titer of serum samples against the alpha, beta, gamma, and delta variants were determined by inhibitory dilution 50 (ID50) by a Pseudovirion-Based Neutralization Assay (PBNA) and reported as reciprocal dilution for each individual sample and GMT for treatment group comparison. The assay was performed at IrsiCaixa AIDS Research Institute (Badalona, Spain), using an HIV based Luciferase reporter pseudovirus pseudotyped with SARS-CoV-2 S protein. Pseudoviruses were generated as described previously^34,35^. For the neutralization assay, 200 TCID50 of pseudovirus supernatant was preincubated with serial dilutions of the heat-inactivated serum samples for 45 min at 37 °C and then added onto Human ACE2 overexpressing HEK293T cells. After 48 h, cells were lysed with britelite plus luciferase reagent (PerkinElmer, Waltham, MA, USA). Luminescence was measured for 0.2 s with an EnSight multimode plate reader (PerkinElmer). The neutralization capacity of the serum samples was calculated by comparing the experimental relative light units (RLUs) calculated from infected cells treated with each serum to the max RLUs (maximal infectivity calculated from untreated infected cells) and min RLUs (minimal infectivity calculated from uninfected cells) and expressed as the neutralization percentage: Neutralization (%) = (RLUmax–RLUexperimental)/(RLUmax–RLUmin) *100. ID50 were calculated by plotting and fitting neutralization values and the log of serum dilution to a 4-parameters equation in Prism 9.0.2 (GraphPad Software, USA).

A validated virus neutralization assay (VNA) was performed using an Alpha SARS-CoV-2 isolate sequenced and deposited in GISAID (ID: EPI_ISL_1663569). Viral-induced cytopathic effect of this VOC preincubated with serial dilutions of serum from vaccinated individuals was measured on Vero E6 cells using the CellTiter Glo Luciferase Cell Viability Assay (Promega). Reciprocal dilutions inhibiting 50% of viral cytopathic effect were calculated as described above. (Detailed methods can be seen in Supplementary Methods 1).

#### Cellular immune response

The T-cell mediated immune responses against the SARS-CoV-2 Spike glycoprotein were assessed on cryopreserved Peripheral Blood Mononuclear Cells (PBMC) by IFN-γ ELISpot and ICS. The cryopreserved PBMCs were thawed in RPMI complemented medium 20% FBS (R20) and then washed two times with RPMI 10% FBS (R10). Cells were counted and plated in a 96-wells round bottom plate using a total of 0.5 × 106 cells per well. Next, PBMCs were stimulated with six peptide pools of overlapping SARS-CoV-2 peptides, each encompassing the SARS-CoV-2 regions S (2 pools) and RBD (4 pools covering Wuhan-Hu-1, alpha, beta, and delta variants), specified below:

•SPIKE_SA: 194 peptides overlapping the S1-2016 to S1-2196 region of the Spike protein from the ancestral Wuhan-Hu-1 strain. •SPIKE_SB: 168 peptides overlapping the S1-2197 to S2-2377 region of the Spike protein from the ancestral Wuhan-Hu-1 strain. •RBD: 84 peptides overlapping the RBD region of the Spike protein (Wuhan-Hu-1 sequence). •RBD_B.1.1.7: 84 peptides overlapping the RBD region of the SARS-CoV-2 alpha variant. •RBD_B.1.351: 84 peptides overlapping the RBD region of the SARS-CoV-2 beta variant. •RBD_B.1617.2: 84 peptides overlapping the RBD region of the SARS-CoV-2 delta variant (this one only applies to ELISpot). PBMC were incubated at a final concentration of 2.5 μg/mL per individual peptide pool. CEF peptide pool (composed of 23 peptides, which are MHC class I-restricted T-cell epitopes from human Cytomegalovirus, Epstein Barr virus and Influenza virus in the concentration 2.0 µg/ml, Mabtech, DK) was also used as positive control. After overnight incubation, each well was washed 6 times with PBS and spot detection was accomplished by a two-step (biotinylated antibody/streptavidin-enzyme) antibody binding process; a 1 h room temperature incubation with biotin plus anti-human IFN-γ, wash 6 times with PBS followed by another 1 h incubation at room temperature with streptavidin. The wells were then incubated with developing solution, followed by 10 min at room temperature with 0.05% Tween 20 in PBS 1X and 6 washes with tap water. After drying upside down, ELISpots were read in the CTL reader system. T-cell responses analyzed by ELISpot were reported as the mean value of spot forming cells per 10^6^ PBMC (SFC/10^6^ PBMC) upon stimulation with each peptide pool, after subtraction of background. In parallel to the spot forming analysis, intracellular staining (ICS) was also performed with PBMC incubated with different peptide pools. Hence, the PBMC were incubated in the presence of 2 μg/mL of monoclonal antibodies against human CD28 (clone L293, BD Pharmingen, catalog number 340450) and CD49d (clone L25, BD Pharmingen, catalog number 340976) for 6 h. During the last 4 h of incubation, GolgiPlug (Brefeldin A, BD Cytofix/Cytoperm Plus, BD Bioscience, catalog number 555028) was added to block cytokine transport. After incubation, PBMC were washed with PBS 1X + 0.5% BSA + 0.1% sodium azide and incubated for 20 min with FcR Blocking Reagent (Milteny Biotec, catalog number 130-059-901, dilution 1:10), then washed and stained for 25 min with the Live/Dead probe (LIVE/DEAD fixable near IR, Thermo Fisher Scientific, catalog number L34975, dilution 1:1000) to discriminate dead cells as well as with surface antigens using the following antibodies: CD3 (SIK7, PerCP, BD Biosciences, catalog number 345766, dilution 1:20), CD4 (clone RPA-T4, BV421, BD Horizon, catalog number 562424, dilution 1:20), CD8 (clone SK1, BV510, BD Horizon, catalog number 563919, 1:40). Afterwards, cells were washed twice in PBS 1X + 0.5% BSA + 0.1% sodium azide, fixed and permeabilized with Fix/Perm kit (BD Cytofix/Cytoperm Plus, BD Biosciences, catalog number 555028) for intracellular cytokine staining. Cells were incubated again for 25 min with FcR Blocking Reagent (Milteny Biotec), washed, and stained with anti-human antibodies of IFN-γ (clone 27, APC, BD Pharmingen, catalog number 554707, dilution 1:20), IL-2 (clone 5344.111, PE, BD FastImmune, catalog number 340450, dilution 1:20) and IL-4 (clone 8D4-8, PECy7, BD Pharmingen, catalog number 560672, dilution 1:20). Finally, stained cells were washed twice with Perm/Wash 1X and fixed in formaldehyde 1%. Cytokine responses were background subtracted. All samples were acquired on BD FACSCanto II (BD Biosciences) flow cytometer and analyzed using FlowJoTM v.10 (10.0.7) software (Tree Star, Inc) using the gating strategy describe in Supplementary fig. 2. ICS assays included Th1/Th2 pathways (e.g., IL-2, IL-4, and IFN-γ) CD4+ and CD8 + T cell determinations using flow cytometry.

### Statistical analysis

Main population analysis, since this Phase 1-2a clinical trial is not confirmatory, it is not possible to justify the sample size numerically in the usual terms of confirmatory trials.

However, the sample size seems reasonable in this exploratory context. With a sample size of *n* = 10 for each 20 µg or 40 µg dose cohort, the probability of observing at least one AE with a prevalence rate of ≥10% is 65.1%. In the 10 µg dose cohort, with a sample size of 10, this probability is 41.0%. For participants in all active groups, *n* = 25, this probability will be 92.8%. These calculations were performed with the nQuery Advisor program version 7.0. Endpoints related to the primary safety outcomes, number of solicited and unsolicited AEs described previously Endpoints related to the primary safety outcomes, number of solicited and unsolicited AEs described previously, as well binary variables related to the immunogenicity, proportion of seroconverted subjects, were described by a proportion and 95% Confidence Interval (95% CI) using exact binomial-based methods and the Clopper-Pearson method^36^. Quantitative results related to immunogenicity and T-cell measurements were analyzed, on previously log-transformed data, using restricted maximum likelihood (REML)-based repeated measures approach (MMRM: Mixed Models for Repeated Measurements). Analyses will include the fixed, categorical effects of group, visit, and group-by-visit interaction. A common unstructured structure was used to model the within-patient correlation. The Kenward-Roger approximation will be used to estimate denominator degrees of freedom^37^. Estimation of effects between and within group were assessed by the ratio and this 95% CI between geometric means. Since this is an exploratory Phase 1/2a trial with no formal interim analysis for early study termination, no alpha adjustments are needed to maintain the type-I error^38^. The statistical software used to analyze all data was SAS version 9.4 (SAS Institute Inc., Cary, NC).

### Reporting summary

Further information on research design is available in the Nature Research Reporting Summary linked to this article.

### Supplementary information

Supplemental Material

reporting summary

## Data Availability

The data that support the findings of this study are available upon request and at the discretion of the Sponsor.

## References

[1] World Health Organization. COVID-19 vaccine tracker and landscape. https://www.who.int/publications/m/item/draft-landscape-of-covid-19-candidate-vaccines

[2] https://www.who.int/news/item/05-05-2023-statement-on-the-fifteenth-meeting-of-the-international-health-regulations-(2005)-emergency-committee-regarding-the-coronavirus-disease-(covid-19)-pand Accessed June 10, 2023.

[3] Looi MK (2021). The world according to covid vaccine coverage. BMJ.

[4] Global Change Data Lab. Our World In Data. Coronavirus (COVID-19) Vaccinations. https://ourworldindata.org/covid-vaccinations. Accessed June 10, 2023.

[5] Lan J (2020). Structure of the SARS-CoV-2 spike receptor-binding domain bound to the ACE2 receptor. Nature.

[6] Greaney AJ (2021). Mapping mutations to the SARS-CoV-2 RBD that escape binding by different classes of antibodies. Nat. Commun..

[7] Greaney AJ (2021). Antibodies elicited by mRNA-1273 vaccination bind more broadly to the receptor binding domain than do those from SARS-CoV-2 infection. Sci. Transl. Med..

[8] Kleanthous H (2021). Scientific rationale for developing potent RBD-based vaccines targeting COVID-19. npj Vacc..

[9] Khoury DS (2021). Neutralizing antibody levels are highly predictive of immune protection from symptomatic SARS-CoV-2 infection. Nat. Med..

[10] Dai L, Gao GF (2021). Viral targets for vaccines against COVID-19. Nat. Rev. Immunol..

[11] Dai L (2020). A Universal Design of Betacoronavirus Vaccines against COVID-19, MERS, and SARS. Cell.

[12] Yang S (2021). Safety and immunogenicity of a recombinant tandem-repeat dimeric RBD-based protein subunit vaccine (ZF2001) against COVID-19 in adults: two randomised, double-blind, placebo-controlled, phase 1 and 2 trials. Lancet Infect. Dis..

[13] Moros A (2023). Immunogenicity and safety in pigs of PHH-1V, a SARS-CoV-2 RBD fusion heterodimer vaccine candidate. Vaccine.

[14] Barreiro A (2023). Preclinical evaluation of a COVID-19 vaccine candidate based on a recombinant RBD fusion heterodimer of SARS-CoV-2. iScience.

[15] Dolgin E (2021). How protein-based COVID vaccines could change the pandemic. Nature.

[16] Keech C (2020). Phase 1–2 Trial of a SARS-CoV-2 Recombinant Spike Protein Nanoparticle Vaccine. N. Eng. J. Med..

[17] Polack FP (2020). Safety and Efficacy of the BNT162b2 mRNA Covid-19 Vaccine. N. Eng. J. Med..

[18] World Health Organization. Tracking SARS-CoV-2 variants. https://www.who.int/activities/tracking-SARS-CoV-2-variants. Accessed June 10, 2023

[19] Menni C (2022). Symptom prevalence, duration, and risk of hospital admission in individuals infected with SARS-CoV-2 during periods of omicron and delta variant dominance: a prospective observational study from the ZOE COVID Study. Lancet.

[20] Abbasi J (2022). Homing In On a SARS-CoV-2 Correlate of Protection. JAMA.

[21] Richmond P (2021). Safety and immunogenicity of S-Trimer (SCB-2019), a protein subunit vaccine candidate for COVID-19 in healthy adults: a phase 1, randomised, double-blind, placebo-controlled trial. Lancet.

[22] Sridhar S (2022). Safety and immunogenicity of an AS03-adjuvanted SARS-CoV-2 recombinant protein vaccine (CoV2 preS dTM) in healthy adults: interim findings from a phase 2, randomised, dose-finding, multicentre study. Lancet Infect. Dis..

[23] He Q (2021). COVID-19 Vaccines: Current Understanding on Immunogenicity, Safety, and Further Considerations. Front. Immunol..

[24] Krammer F (2021). A correlate of protection for SARS-CoV-2 vaccines is urgently needed. Nat. Med..

[25] Khoury DS (2023). Correlates of Protection, Thresholds of Protection, and Immunobridging among Persons with SARS-CoV-2 Infection. Emerg. Infect. Dis..

[26] Tarke A (2022). SARS-CoV-2 vaccination induces immunological T cell memory able to cross recognize variants from Alpha to Omicron. Cell.

[27] Tarke A, Grifoni A, Sette A (2022). Bioinformatic and experimental analysis of T cell immune reactivity to SARS CoV-2 and its variants. Front. Bioinform..

[28] Tarke A (2021). Impact of SARS CoV- 2 variants on the total CD4(+) and CD8(+) T cell reactivity in infected or vaccinated individuals. Cell Rep. Med..

[29] Zhang Z (2022). Humoral and cellular immune memory to four COVID19 vaccines. Cell.

[30] Corominas J (2023). Safety and immunogenicity of the protein-based PHH-1V compared to BNT162b2 as a heterologous SARS-CoV-2 booster vaccine in adults vaccinated against COVID-19: a multicentre, randomised, double-blind, non-inferiority phase IIb trial. Lancet Reg. Health Eu.

[31] European Medicines Agency. News. https://www.ema.europa.eu/en/news/ema-recommends-approval-bimervax-covid-19-booster-vaccine. Accessed June 10, 2023.

[32] European Medicine Agency. Science Medicine Health. Cominarty. https://www.ema.europa.eu/en/medicines/human/EPAR/comirnaty. Accessed June 10, 2023.

[33] European Medicines Agency. Guideline for good clinical oractive E6 (R2). https://www.ema.europa.eu/en/documents/scientific-guideline/ich-guideline-good-clinical-practice-e6r2-step-5_en.pdf. Accessed June 10, 2023.

[34] Pradenas E (2021). Stable neutralizing antibody levels 6 months after mild and severe COVID-19 episodes. Med.

[35] Pradenas E (2022). Clinical course impacts early kinetics, magnitude, and amplitude of SARS-CoV-2 neutralizing antibodies beyond 1 year after infection. Cell Rep. Med..

[36] Agresti A. *Categorical Data Analysis*. 2nd Edition, (John Wiley & Sons, Inc; New York, 2002).

[37] Mallinckrod CH, Lane PW, Schnell D, Peng Y, Mancuso JP (2008). Recommendations for the Primary Analysis of Continuous Endpoints in Longitudinal Clinical Trials. Ther. Innov. Regul. Sci..

[38] The European Agency for the Evaluation of Medicinal Products. CPMP/EWP/908/99. Points to Consider on Multiplicity issues in Clinical Trials. URL: http://www.ema.europa.eu/ema/index.jsp?curl=pages/regulation/general/general_content_001220.jsp&mid=WC0b01ac05807d91a4 Accessed July 14 2022.

