# Supplementary material for: Safety and immunogenicity of a recombinant protein RBD fusion heterodimer vaccine against SARS-CoV-2
Source: NPJ Vaccines. 2023 Sep 29;8:147. doi: 10.1038/s41541-023-00736-5 (PMC10541407; doi:10.1038/s41541-023-00736-5)

SUPPLEMENTARY SECTION

HIPRA-HH-1V study group

- Hospital Clínic Barcelona, IDIBAPS, Universitat de Barcelona, Barcelona Spain: Eva Bonfill, Omar Anagua, Faisury Caicedo, Clara Castán, Fauno Guazina, Sara Messeguer, Marta Aldea, Anna Vilella, Sandra Serrano, Lorna Leal PhD, Judit Pich, Jocelyn Nava, Karla Escobar, Joan Albert Arnaiz, Alex Soriano, José Ríos, Teresa Botta, Ignasi Esteban, Carmen Pastor, Montserrat Plana, Gemma Domenech.

- IRSICAIXA: Silvia Marfil, Carla Rovirosa, Raquel Ortiz, Daniel Perez-Zsolt, Marçal Gallemí, Edwards Pradenas, Dàlia Raïch-Regué, Benjamin Trinité, Jordana Muñoz-Basagoiti, Bonaventura Clotet, Nuria Izquierdo-Useros, Julià Blanco.

- Institut Universitari d'Investigació en Atenció Primària Jordi Gol (IDIAP Jordi Gol), Biomedical Research Institute (IdIBGi), Girona, Spain: Marina González del Río Ruth Martí-Lluch Marc Ribas-Aulinas Aida Corpes-Comes, Marga Nadal, Rafel Ramos.

- HIPRA: Luís González, Manuel Cañete, Laia Madrenas, Alexandra Moros, Irina Güell, Laura Ferrer, Antoni Prenafeta, Júlia Corominas, Carme Garriga, Antonio Barreiro, Teresa Prat, Elia Torroella.

Supplementary figure 1

Total SARS-CoV-2 specific T-cell responses measured by IFN-γ ELISPOT against variants of concern (VOC) alpha (B.1.1.7), beta (B.1.351), delta (B.1.617.2) and RBD peptide mix variants, at screening, 21 and 35 days after first vaccination and 12 weeks after second vaccination are shown for all participants distributed by vaccine group PHH-1V 10µg n= 5, PHH-1V 20µg n=10, Cohort 3 PHH-1V 40µg n=10, control n=5. T-cell responses are expressed as mean spot forming cells / 10^6 PBMC. Bars indicate 95% confidence intervals and data points represent individual results.

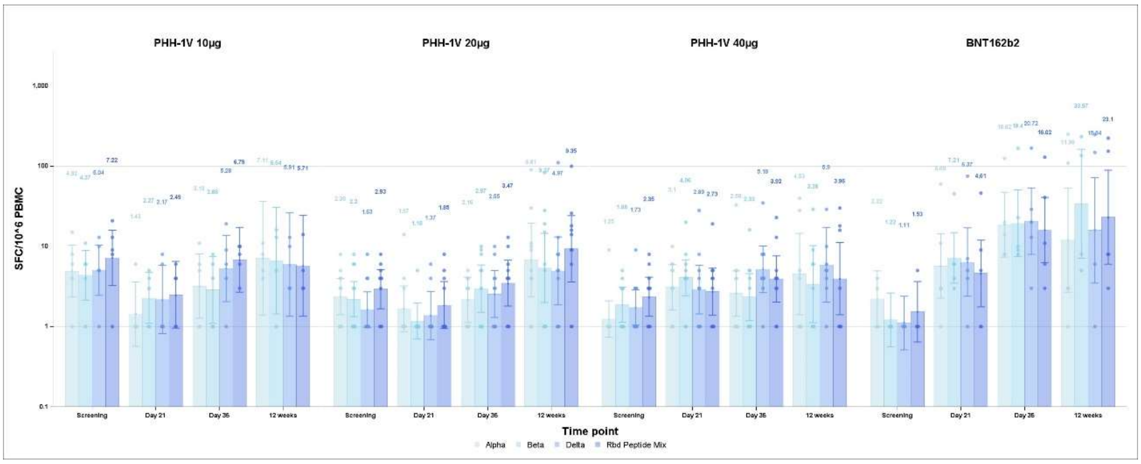

Supplementary Table 1

Humoral and cellular immunogenicity data censored obtained at 24W and 48W visits.

Censored data on total binding antibodies titres measured by ELISA at 24W and 48W time-points are shown by vaccine group: 24W PHH-1V 10µg n=2, PHH-1V 20µg n=6, Cohort 3 PHH-1V 40µg n=4, control n=4; 48W PHH-1V 10µg n=1, PHH-1V 20µg n=3, Cohort 3 PHH-1V 40µg n=3, control n=3. Total binding antibodies titers are expressed in GMT shown as adjusted treatment mean (95% CI). GMT geometric mean titer; CI confidence interval.

Neutralization antibodies titres measured by pseudovirus-based neutralization assay (PBNA) against variants of concern assessed alpha (B.1.1.7), beta (B.1.351), delta (B.1.617.2) and gamma (P.1) at 24W and 48W time-points are shown by vaccine group: 24W PHH-1V 10µg n=2, PHH-1V 20µg n=6, Cohort 3 PHH-1V 40µg n=4, control n=4; 48W PHH-1V 10µg n=1, PHH-1V 20µg n=3, Cohort 3 PHH-1V 40µg n=3, control n=3. Neutralization titers measured as IC_{50} are expressed in GMT shown as adjusted treatment mean (95% CI).

T-cell responses measured by IFN-γ ELISPOT against variants of concern assessed alpha (B.1.1.7), beta (B.1.351), delta (B.1.617.2) and RBD peptide mix at 24W time-point are shown by vaccine group: PHH-1V 10µg n=2, PHH-1V 20µg n=6, Cohort 3 PHH-1V 40µg n=4, control n=4. T cell response is expressed as mean spot forming cells / 10^6 PBMC (95% CI).

	PHH-1V 10µg	PHH-1V 20µg	PHH-1V 40µg	BNT162b2
Binding Antibodies				
24 Week	(n=2)<br>981.01<br>(101.55 - 9476.81)	(n=6)<br>392.21<br>(84.51 - 1820.2)	(n=3)<br>4370.95<br>(804.39 - 23751.11)	(n=4)<br>1837.21<br>(232.97 - 14488.49)
48 Week	(n=1)<br>927.55<br>(87.15 - 9872.42)	(n=3)<br>1717.39<br>(401.39 – 7348)	(n=3)<br>2440.96<br>(407.61 - 14617.57)	(n=3)<br>1955.56<br>(322.83 - 11845.87)
Neutralizing Antibodies				
ALPHA B.1.1.7				
24 Week	(n=2)<br>167.41<br>(13.43 - 2086.51)	(n=6)<br>104.52<br>(20.71 - 527.52)	(n=3)<br>2979.36<br>(419.78 – 21145.74)	(n=4)<br>375.29<br>(46.18 – 3049.67)
48 Week	(n=1)<br>586.37<br>(73.7 - 4665.25)	(n=3)<br>669.75<br>(196.92 - 2277.88)	(n=3)<br>1385.86<br>(268.87 - 7143.14)	(n=3)<br>429.62<br>(95.26 - 1937.49)
BETA B.1.351				
24 Week	(n=2)<br>250.84<br>(17.79 -3537.15)	(n=6)<br>139.97<br>(25.14 - 779.29)	(n=3)<br>2902.13<br>(376.43 - 22374.29)	(n=4)<br>559.57<br>(59.92 - 5225.64)
48 Week	(n=1)<br>250.91<br>(34.68 - 1815.5)	(n=3)<br>856.35<br>(261.77 - 2801.41)	(n=3)<br>4991.44<br>(1027.14 - 24256.2)	(n=3)<br>801.07<br>(181.61 – 3533.44)
DELTA B.1.617.1				
24 Week	(n=2)<br>294.19<br>(27.53 - 3144.12)	(n=6)<br>64.74<br>(15.61 - 268.45)	(n=3)<br>10143.23<br>(1518.42 - 67758.2)	(n=4)<br>413.47<br>(68.9 - 2481.06)
48 Week	(n=1)<br>215.25<br>(47.74 - 970.62)	(n=3)<br>110.8<br>(45.29 - 271.05)	(n=3)<br>978.46<br>(305.79 - 3130.85)	(n=3)<br>356.31<br>(118.7 - 1069.57)
GAMMA P.1				
24 Week	(n=2)<br>348.09<br>(26.6 - 4555.03)	(n=6)<br>125.3<br>(26.08 - 602)	(n=3)<br>4562.92<br>(589.43 - 35322.89)	(n=4)<br>480.87<br>(65.63 - 3523.35)
48 Week	(n=1)<br>644.05<br>(72.46 - 5724.85)	(n=3)<br>362.42<br>(100.78 - 1303.26)	(n=3)<br>3058.97<br>(524.22 - 17849.91)	(n=3)<br>560.44<br>(114.77 - 2736.8)
T-cell responses				
ALPHA B.1.1.7				
24 Week	(n=2)<br>5.32<br>(0.24 - 117.38)	(n=6)<br>2.11<br>(0.25 - 17.49)	(n=3)<br>77.41<br>(7.16 - 837.38)	(n=4)<br>79.49<br>(4.11 - 1536.36)
BETA B.1.351				
24 Week	(n=2)<br>0.63<br>(0.09 - 4.27)	(n=6)<br>7.17<br>(2.07 - 24.81)	(n=3)<br>27.55<br>(5.72 - 132.8)	(n=4)<br>100.87<br>(16.51 - 616.32)
DELTA B.1.617.1				
24 Week	(n=2)<br>15.48<br>(4.01 - 59.73)	(n=6)<br>5.54<br>(2.39 - 12.8)	(n=3)<br>17.05<br>(5.24 - 55.52)	(n=4)<br>41.29<br>(12.38 - 137.74)
RBD PEPTIDE MIX				
24 Week	(n=2)<br>8.85<br>(1.17 - 67.23)	(n=6)<br>5.62<br>(1.67 - 18.93)	(n=3)<br>24.91<br>(3.54 - 175.04)	(n=4)<br>34.44<br>(7.14 - 166.1)

Supplementary methods 1

Biosafety Approval.

The biologic biosafety committee of the Research Institute Germans Trias i Pujol approved the execution of SARS-CoV-2 experiments at the BSL3 laboratory of the Center for Bioimaging and Comparative Medicine (CSB-20-015-M3).

Cell culture and Viral isolation and titration.

Vero E6 cells (ATCC CRL-1586) were cultured in Dulbecco's modified Eagle medium (Invitrogen) supplemented with 10% fetal bovine serum (FBS; Invitrogen), 100 U/ml penicillin, 100 µg/ml streptomycin, (all from Invitrogen).

SARS-CoV-2 was isolated from a nasopharyngeal swab collected in January 2021 in Spain in Vero E6 cells as described in (1). The virus stock was prepared collecting the supernatant from Vero E6 and sequenced as detailed in (1). Genomic sequence was deposited at GISAID repository (http:// gisaid.org) with accession number EPI_ISL_1663567. Viral stocks were propagated in Vero E6 cells for two passages and titrated in 10-fold serial dilutions to calculate the $TCID_{50}$ per mL. Infection was set to achieve a 50% of viral induced cytopathic effect measured with Cell Titer GloTM Luciferase reagent, as described in the next section.

Neutralization Assay.

Neutralization assays were performed in duplicate. Briefly, 60 $TCID_{50}$ of SARS-CoV-2 were preincubated with serial dilutions of heat-inactivated plasma samples from the indicated individuals for 1 h at 37 ºC. Pre-incubated viruses were added to 60.000 Vero E6 cells per well in 96 well plates. 72 h later, viral-induced cytopathic effect was measured using the Cell Titer Glo Luciferase reagent (Promega) and the Luminoskan Plate Reader from Thermofisher. The relative light units (RLU) were normalized to untreated non-infected cells (without plasma or virus), and the ID_{50} (the reciprocal dilution inhibiting 50% of the cytopathic effect) was calculated by plotting and fitting the log of plasma dilution vs. response to a 4-parameter equation in GraphPad Prism 9.3.1, as previously described in (2, 3, 4).

1. Rodon, J., Muñoz-Basagoiti, J., Perez-Zsolt, D., Noguera-Julian, M., Paredes, R., Mateu, L., Quiñones, C., Perez, C., Erkizia, I., Blanco, I., Valencia, A., Guallar, V., Carrillo, J., Blanco, J., Segalés, J., Clotet, B., Vergara-Alert, J., Izquierdo-Useros, N., 2021. Identification of Plitidepsin as Potent Inhibitor of SARS-CoV-2-Induced Cytopathic Effect After a Drug Repurposing Screen. Front. Pharmacol. 12, 646676. https://doi.org/10.3389/fphar.2021.646676

2. Trinité B, Tarrés-Freixas F, Rodon J, Pradenas E, Urrea V, Marfil S, Rodríguez de la Concepción ML, Ávila-Nieto C, Aguilar-Gurrieri C, Barajas A, et al. SARS-CoV-2 infection elicits a rapid neutralizing antibody response that correlates with disease severity. *Sci Rep* (2021) 11:2608. doi:10.1038/s41598-021-81862-9

3. Trinité B, Pradenas E, Marfil S, Rovirosa C, Urrea V, Tarrés-Freixas F, Ortiz R, Rodon J, Vergara-Alert J, Segalés J, et al. Previous SARS-CoV-2 Infection Increases B.1.1.7 Cross-Neutralization by Vaccinated Individuals. *Viruses* (2021) 13:1135. doi:10.3390/v13061135

4. Pradenas E, Trinité B, Urrea V, Marfil S, Ávila-Nieto C, Rodríguez de la Concepción ML, Tarrés-Freixas F, Pérez-Yanes S, Rovirosa C, Ainsua-Enrich E, et al. Stable neutralizing antibody levels six months after mild and severe COVID-19 episode. *Med* (2021)S2666634021000350. doi:10.1016/j.medj.2021.01.005

Supplementary Figure 2

Example of gating strategy used for the analysis of intracellular cytokine stainings (ICS) from peripheral blood mononuclear cells (PBMCs). First, total cells were selected excluding cellular debris (SSC-A vs FSC-H). Then single cells were separated from doublets (FSC-H vs FSC-A). Next, CD3 positive cells were selected excluding autofluorescent and CD3 negative cells (Empty vs CD3-PerCp). After, CD3 postive living cells were determined (live/dead vs CD3-PerCp) and then CD4 T-cells and CD8 T-cells were selected (CD4-BV421 vs CD8-BV510). Finally, CD4 and CD8 T-cells positive for IL-2, IFNγ and IL-4 were determined (IFNγ-APC vs IL-2-PE) and (IFNγ-APC vs IL-4-PE-Cy7).

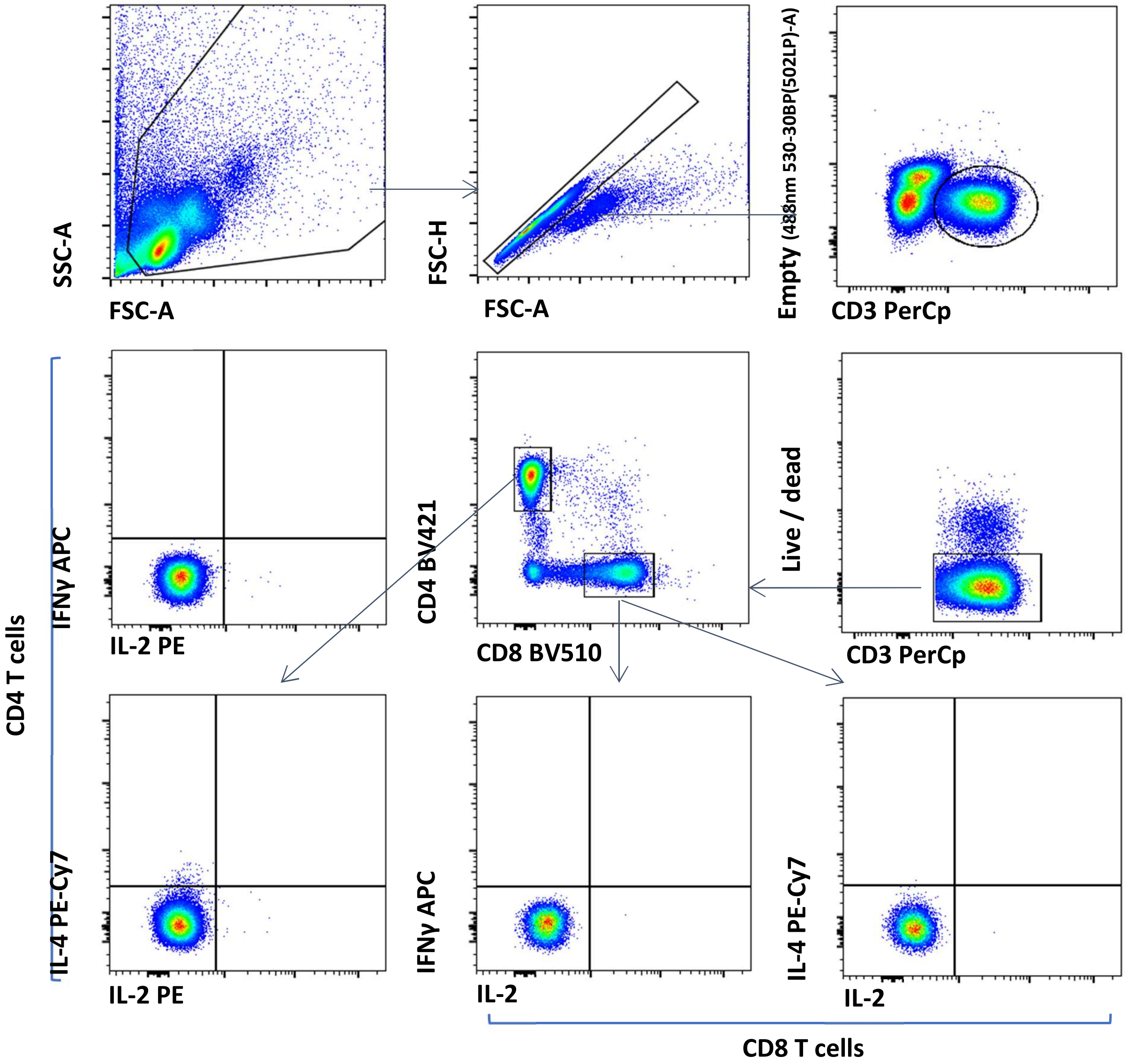

Supplement: Supplementary file 1 — Supplemental Material [file 41541_2023_736_MOESM1_ESM.pdf]
